# Testing frailty, accessible housing, and changes in living arrangements using the Health and Retirement Study

**DOI:** 10.1093/geroni/igab046.2365

**Published:** 2021-12-17

**Authors:** Bonnie Albright

**Affiliations:** University of Massachusetts - Boston, Boston, Massachusetts, United States

## Abstract

This study examined housing accessibility elements of community-dwelling older adults using the Health and Retirement Study (HRS). Housing accessibility elements were tested as moderators in the relationship between prior frailty and later living arrangements. HRS physical measures were used to construct the Physical Frailty Phenotype and the Continuous Frailty Scale. The analytic method for the study was multinomial logistic regression. Latent class analysis was also used to identify housing accessibility element use-types. Study findings will be presented. Strengths and weaknesses of using the HRS to measure home accessibility and construct frailty scales will also be discussed.

